# Ultra-Broadband Acoustic Diode in Open Bend Tunnel by Negative Reflective Metasurface

**DOI:** 10.1038/s41598-018-34314-w

**Published:** 2018-10-31

**Authors:** Qingxuan Liang, Yong Cheng, Jin He, Jinke Chang, Tianning Chen, Dichen Li

**Affiliations:** School of Mechanical Engineering and State Key Laboratory for Manufacturing Systems Engineering, Xi’an Jiaotong University, Xi’an, 710049 People’s Republic of China

## Abstract

We theoretically and numerically propose an open bend tunnel with the capability of realizing ultra-broadband unidirectional transmission. The designed tunnel can isolate acoustic wave incidence from opposite directions and substance like the fluids or objects can exchange freely by employing acoustic gradient metasurface. The underlying mechanism is due to apparent negative reflection in ultra-broadband frequency range when the incoming angle impinging on the metasurface is over the critical incidence. The numerical results keep a good agreement with the theoretical analyses. The proposed design could be employed to generate various situations, like broadband noise control, architectural acoustics and ultrasound imaging.

## Introduction

Researching acoustic diode (AD) technology has become an interesting and challenging problem in both scientific and engineering communities since sound has been thought to propagate easily along two opposite directions in any path. Acoustic diode can realize unidirectional sound manipulation in a subwavelength space and possesses many potential applications^1–6^. Although researchers have achieved remarkable progresses in both linear and non-linear regimes by using various structures, such as grating structures^7,8^, phononic crystals^9,10^ or metamaterials^3,11^, the resulting ADs are only designed to have a narrow and limited asymmetric transmission bandwidth. A broader asymmetric transmission bandwidth is particularly favorable so as to realize one-way wave steering in a wide frequency range for AD applications. Recently gradient metasurface has attracted considerable interests owning to the unusual ability to steer waves arbitrarily with a planar subwavelength structure^12–21^. Cheng *et al*. demonstrate a four-body periodic Helmholtz resonator array to realize one-way sound propagation in a broadband range by employing acoustic metasurfaces^22^. However, the designed structure is relatively complex for AD applications. Wang *et al*. propose a wideband sound one-way device with impedance-matched sound metasurfaces^23^. However, the design has an obstacle to complexity in experimental realization. Apparently a new mechanism is required to explore effectively to how to broaden the work frequency bandwidth.

Based on the generalized refractive and reflective law, a critical condition that the abnormal refraction and reflection can occur^24–28^, and the incoming wave would be converted into the surface wave along the metasurface. Recent studies report the result of surface negative refraction and negative reflection **w**hen the incoming angle impinging on the metasurface is over the critical incidence^27,29^. Jiang *et al*. investigate the surface negative reflective property that happens when the incoming angle impinging on the metasurface is over the critical incidence, in which no diffractive order reflects owning to the generalized reflective law, can be implemented by a reflective periodic phase-modulating metasurface^29^. Therefore, it may offer an effective approach to design acoustic one-way devices by combining abnormal reflection and surface negative reflective performance of acoustic metasurface^30–32^. However, previous work only reveals the influence of the incident angle on the reflective performance of acoustic metasurface^26^. The influence of incident frequency on negative reflection is still absent so far when the incoming wave impinging on the metasurface over the critical angle.

In this present work, ultra-broadband asymmetric transmission in open bend tunnel by apparent negative reflective metasurface is theoretically designed and numerically demonstrated. Meantime, according to joining periodic phase-steering structure factor into the generalized reflective law^29^, negative reflection will take place in a broadband frequency range. The theoretical research indicates that the apparent negative reflection can occur in a broadband frequency range beyond the critical angle domain. The numerical results have a good agreement with the theoretical analyses. The extraordinary character from negative reflective metasurface has immense wave manipulation capability in a broadband frequency range and could offer a new way to design one- way sound steering devices, and possess potential applications in various situations, such as broadband noise control, architectural acoustics and ultrasound imaging.

## Results

**Figure 1:**
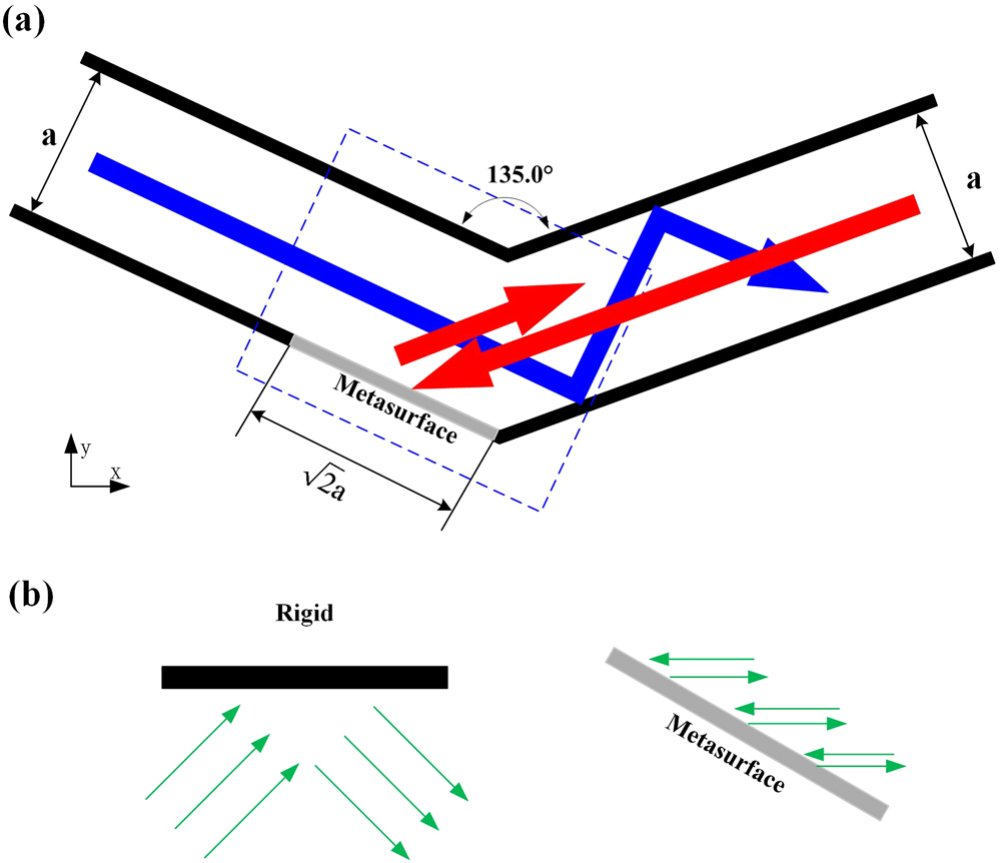
(**a**) Sketch of sound one-way gradient acoustic metasurface tunnel. (**b**) Sketch of the unique transmission of the designed model. For rigid boundaries, there is a normal reflector. However, the apparent negative reflective metasurface plays the crucial role that finally results in the asymmetric propagation.

The simple model is used to realize an acoustic one-way device, as the two-dimensional (2D) schematic diagrams shown in Fig. 1(a). The model is a combination of the gradient acoustic metasurface and rigid boundaries. In this paper, unblocked direction (UD) is defined as the direction that the incident plane wave is allowed to pass the bend tunnel, represented by blue arrows in Fig. 1(a). The incident plane wave direction of blocked passageway (red arrows) is called blocked direction (BD). The width of tunnel is a, the length of the acoustic metasurface is $\sqrt{2}{\rm{a}}\,$, and the bending angle is 135°. When a plane acoustic wave incident from the left port, the propagated wave impinges on the rigid boundaries with normal reflection and passes the bend tunnel freely because the width of tunnel is much larger than the work wavelength, as the blue arrows illustrate. Instead, for right incidence, the transmitted beam will be reflected due to negative reflective metasurface, like showed in the red arrows. Gradient acoustic metasurface plays a crucial role in reflection wave-steering capability, which is employed against the conversion symmetry between incident waves and reflection waves on the surface. As shown in Fig. 1(b), the incident wave is reflected normally for a rigid boundary. However, the transmitted acoustic wave is directly reflected to the incoming port by negative reflection of acoustic metasurface and forbidden to pass.

**Figure 2:**
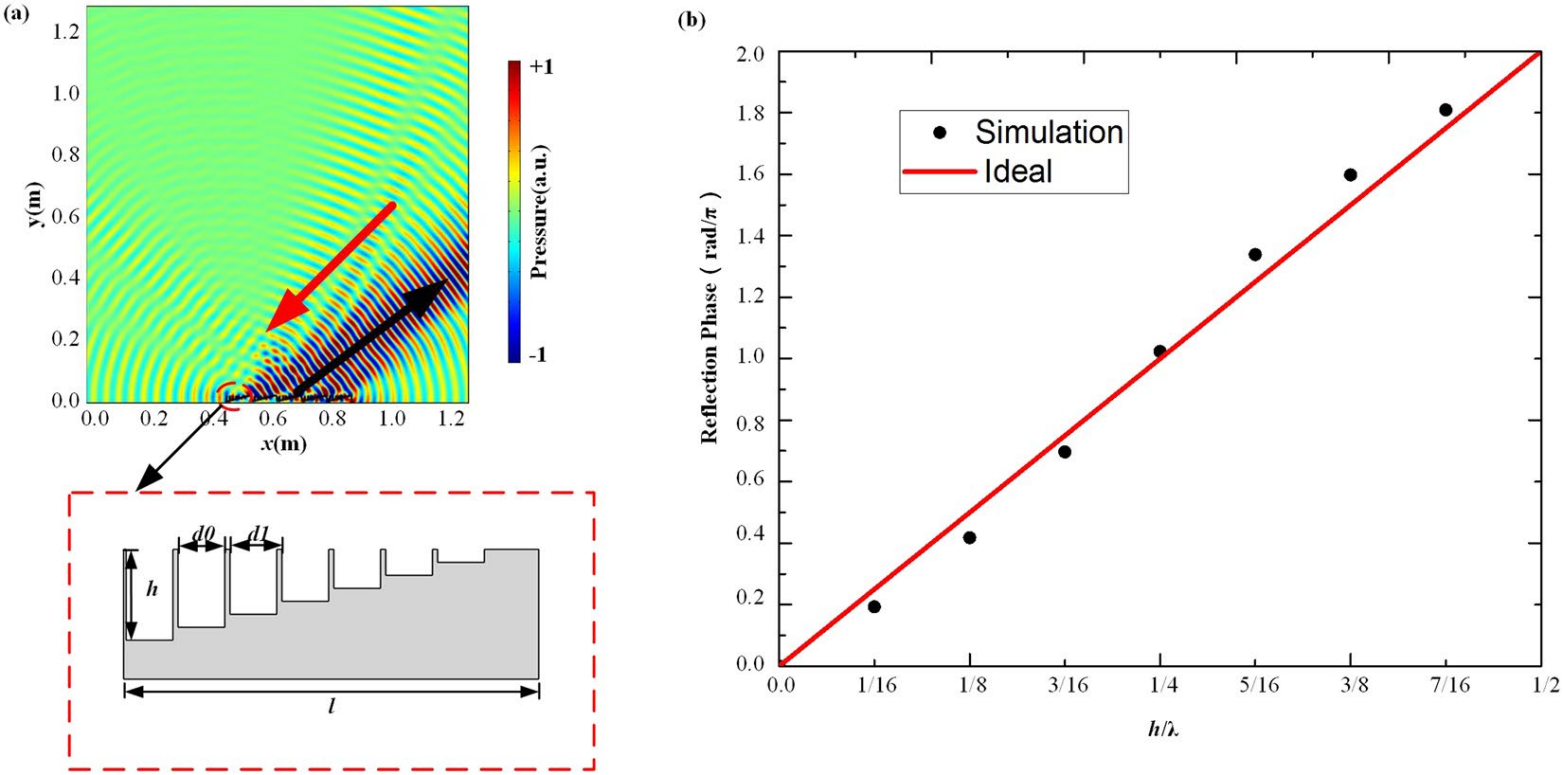
(**a**) The reflection pressure field distribution for the oblique −45° plane acoustic wave incident on the acoustic metasurface with finite length 6 *l* at 8575 Hz and the schematic of an individual period. (**b**) The ideal phase shifts (red line) and the discrete phase gradient provided by acoustic metasurface (black dots) of the grooves as a function of $h/\lambda$ at 8575 Hz.

To obtain the desired negative reflection, the full 2π range has been covered on the interface of the reflection phase response. The gradient acoustic metasurface is designed with finite thick solid plates to separate the adjacent grooves^33^. When plane wave is normally incident on the unit at the designed frequency of *f*_*0*_ = 8575 Hz, the relationship between the phase shifts at the interface and the depths of the grooves is simply determined by the wave path, $\nabla {\rm{\phi }}=\frac{-4\pi h}{\lambda }$, as shown in Fig. 2(b). The numerical results keep a good agreement with the theoretical predictions. As shown in Fig. 2(a), a supercell of acoustic metasurface consists of eight units with the gradient depths *h* ranging from 0 to $7\lambda /16$ with step of $\lambda /16$ (${\rm{\lambda }}$ is the work wavelength). Based on the generalized reflective law, the acoustic wave will change path in the subwavelength thickness tubes. We can realize the expected phase gradient metasurface with a suitable period of the supercell (*l* = 8*d*_1_). Here, the period of unit is *d*_1_ = 10 mm, the width of the groove is *d*_0_ = 8 mm, the period of supercell is *l* = 80 mm. When the oblique −45° plane acoustic wave incidents on the metasurface, according to formula (2), the reflection angle $\,{\theta }_{re}$ is 52.4°, as shown in Fig. 2(a), corresponding to ${n}_{G}=-\,4$, ${d}_{1}=\lambda /4$. The width of metasurface array consisting of six supercells is 480 mm of $\sqrt{2}{\rm{a}}$, the width of bend waveguide in Fig. 2(a) is much larger than the practical wavelength in the frequency.

The reflective metasurface offers a discontinues phase-steering covering 2π range in a supercell period ($l$), and this phase gradient term follows ${\rm{\xi }}={\rm{\sigma }}\frac{d\phi }{dx}=\sigma \frac{2\pi }{l},\,\sigma =1\,{\rm{or}}\,-\,1$, which indicates the direction of the surface phase gradient, $\frac{d\phi }{dx}$ is phase gradient on the metasurface along x-axis. The metasurface’s reflected performance on the premise that the incident waves are plane waves is described as the generalized law of reflection^14^

1 $$(\sin \,{\theta }_{re}-\,\sin \,{\theta }_{i}){k}_{0}=\xi \,$$

where ${\theta }_{i}$ is incident angle, ${\theta }_{re}$ is reflected angle, $\xi$ is phase gradient along the direction of the surface, ${k}_{0}=\frac{2\pi }{\lambda }$ is wave number in the air,$\,{\rm{\lambda }}$ is wavelength. Here, the incident critical angles $\,{\theta }_{c}={\rm{sgn}}(\xi )\arcsin (1-|{k}_{s}|)$ of the generalized reflective law could be solved theoretically, ${k}_{s}=\frac{\xi }{{k}_{0}}$ denotes the reduced surface gradient. When the incoming angle impinging on the metasurface is over the critical incidence, high-order waves would be excited owing to periodicity of the structure instead of being converted to surface wave as predicted by Equation (1). To theoretical prediction about full-angle incident item, the generalized reflective law could be expressed as^24^

2 $$(\sin \,{\theta }_{re}-\,\sin \,{\theta }_{i}){k}_{0}=(1+{n}_{G})\xi \,$$

where ${n}_{G}$ indicates the corresponding diffraction order. In this work, the phase gradient is set as ${\rm{\xi }}=-\,\frac{2\pi }{l}$, the incident critical angles ${\theta }_{c}=-\,\arcsin (1-\frac{\lambda }{l})$ could be determined theoretically. For the classical diffraction theory, when incoming angle is below the critical incidence, ${n}_{G}$ = 0. When the incoming angle impinging on the metasurface is over the critical incidence, the incident frequency is a key factor in reflected behaviors of the reflection phase gradient metasurfaces for the formula (2). The generalized reflective law could be expressed as

3 $$\sin \,{\theta }_{re}-\,\sin \,{\theta }_{i}=(1+{n}_{G}){k}_{s}\frac{{f}_{0}}{f}\,$$

where *f* is the incident frequency, *f*_*0*_ is the designed frequency of phase gradient metasurface. As an analogue of the incident critical angle, the incident critical frequency ${f}_{c,{n}_{G}}=\frac{(1+{n}_{G})}{(1-\,\sin \,{\theta }_{i})}{k}_{s}{f}_{0}$ can be deduced mathematically by formula (3). The critical frequency and angle defines together the domain that satisfies the momentum matching condition and the non-local effect along the metasurface. According to formula (2), when incoming angle is outside the incident critical angles range, *n*_*G*_ would support the equation with a corresponding frequency. In this case, by selecting a proper reflected gradient metasurface, desired broadband negative reflection can be achieved.

**Figure 3:**
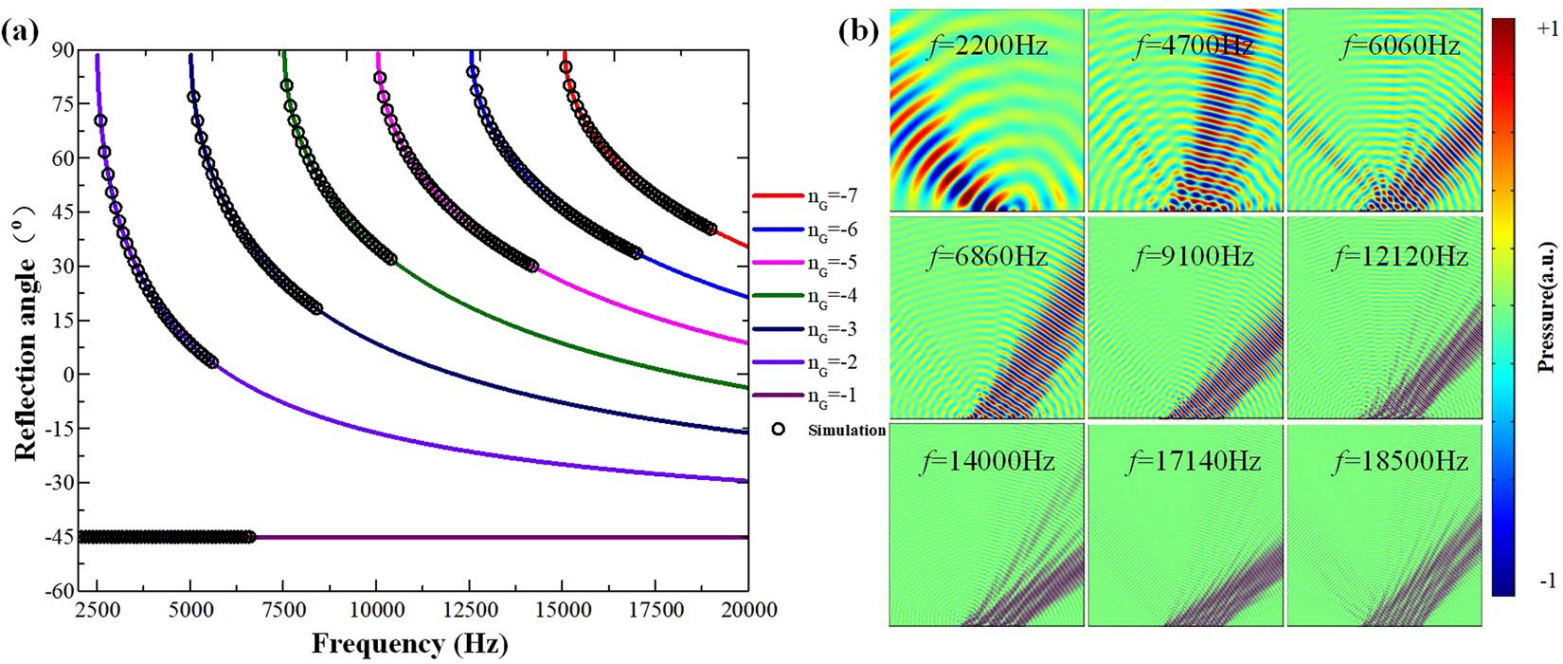
The reflective behaviors when the wave incident at oblique −45° on the metasurface of phase gradient $-0.5{k}_{0}$. (**a**) The influence of incoming frequency on reflected angle. (**b**) Calculated scattered acoustic field of different incident frequency at 2200 Hz, 4700 Hz, 6060 Hz, 6860 Hz, 9100 Hz, 12120 Hz, 14000 Hz, 17140 Hz, 18500 Hz the beam reflect at −45°, 12°, 45°, 33°, 45°, 45°, 55°, 52°and 43°, correspondingly.

We investigate the influence of incident frequency on negative reflective performance of metasurface in a broadband range when the incoming angle impinging on the metasurface is over the critical incidence. The acoustic metasurface consists of 6 supercells in this simulation. Owing to the wave vector component of the incoming wave along the surface of metasurface ${k}_{\parallel }=\frac{2\pi {c}_{air}}{f}\,\sin \,{\theta }_{i}$ is incoming angle and frequency dependent, the value of ${n}_{G}$ would be frequency dependent when the incoming angle is invariant. When the incident frequency is beyond the critical frequency from the formula (3), the value of ${n}_{G}$ follows the ‘jump-up’ rule^26^. Previous research of the gradient metasurface shows that the corresponding diffraction order $|{n}_{G}$ is −4, for surface phase gradient *−*0.5 ${k}_{0}$, when incident angle is *θ*_*i*_ = −45° ^26^. Figure 3 shows the reflective performance of the metasurface when phase gradient is *−*0.5 ${k}_{0}$ in the frequency range from 2000 Hz to 19000 Hz. In this case, the critical frequency ${f}_{c,{n}_{G}}\,\,$of the two adjacent reflection states is 2511.6 Hz, 5023.1 Hz, 7534.7 Hz, 10046.2 Hz, 12557.8 Hz, 15069.4 Hz, can be calculated by ${f}_{c,{n}_{G}}=\frac{(1+{n}_{G})}{(1-\,\sin \,{\theta }_{i})}{k}_{s}{f}_{0}$, corresponding seven possible reflection states $|{n}_{G}\rangle$: $|-1\rangle$, $|-2\rangle$, $|-3\rangle$, $|-4\rangle$, $|-5\rangle$, $|-6\rangle$ and $|-7\rangle$. When *f* < 2511.6 Hz, the reflected angle can be calculated by ${\theta }_{re}={\theta }_{i}$, the reflection states ${n}_{G}$ is −1, as the purple solid line shown in Fig. 3(a). Similarly, when 2511.6 Hz < *f* < 5023.1 Hz, 5023.1 Hz < *f* < 7534.7 Hz, 7534.7 Hz < *f* < 10046.2 Hz, 10046.2 Hz < *f* < 12557.8 Hz, 12557.8 Hz < *f* < 15069.4 Hz, 15069.4 Hz < *f* < 19000 Hz and the corresponding reflective angle can be achieved by ${\theta }_{re}=\arcsin [sin{\theta }_{i}+(1+{n}_{G}){k}_{s}\frac{{f}_{0}}{f}]$. Figure 3(a) shows the relationship between reflected angle and incident frequency. The corresponding diffraction orders of incident frequency have a good agreement with the numerical results by Equation (3). The simulated reflective acoustic pressure field of different incoming frequencies is as shown in Fig. 3(b). When the incident plane sound wave frequency is 2200 Hz, 4700 Hz, 6060 Hz, 6860 Hz, 9100 Hz, 12120 Hz, 14000 Hz, 17140 Hz and 18500 Hz respectively, the beam reflects at −45°, 12°, 45°, 33°, 45°, 45°, 55°, 52° and 43°, correspondingly. The results agree with the theoretical reflected angle of −45°, 11.8°, 45.1°, 32.9°, 44.9°, 45.1°, 55.5°, 52.5° and 43.1°. The simulation results demonstrate that negative reflection can be maintained in an ultra-broadband frequency range and the typical results will be presented as follows by the acoustic metasurface, which plays an important role in the acoustic one-way device to realize the AD effect. (Detailed discussion about the influence of the different phase gradients of acoustic gradient metasurface on the negative reflection behaviors is presented in Supplementary Information).

**Figure 4:**
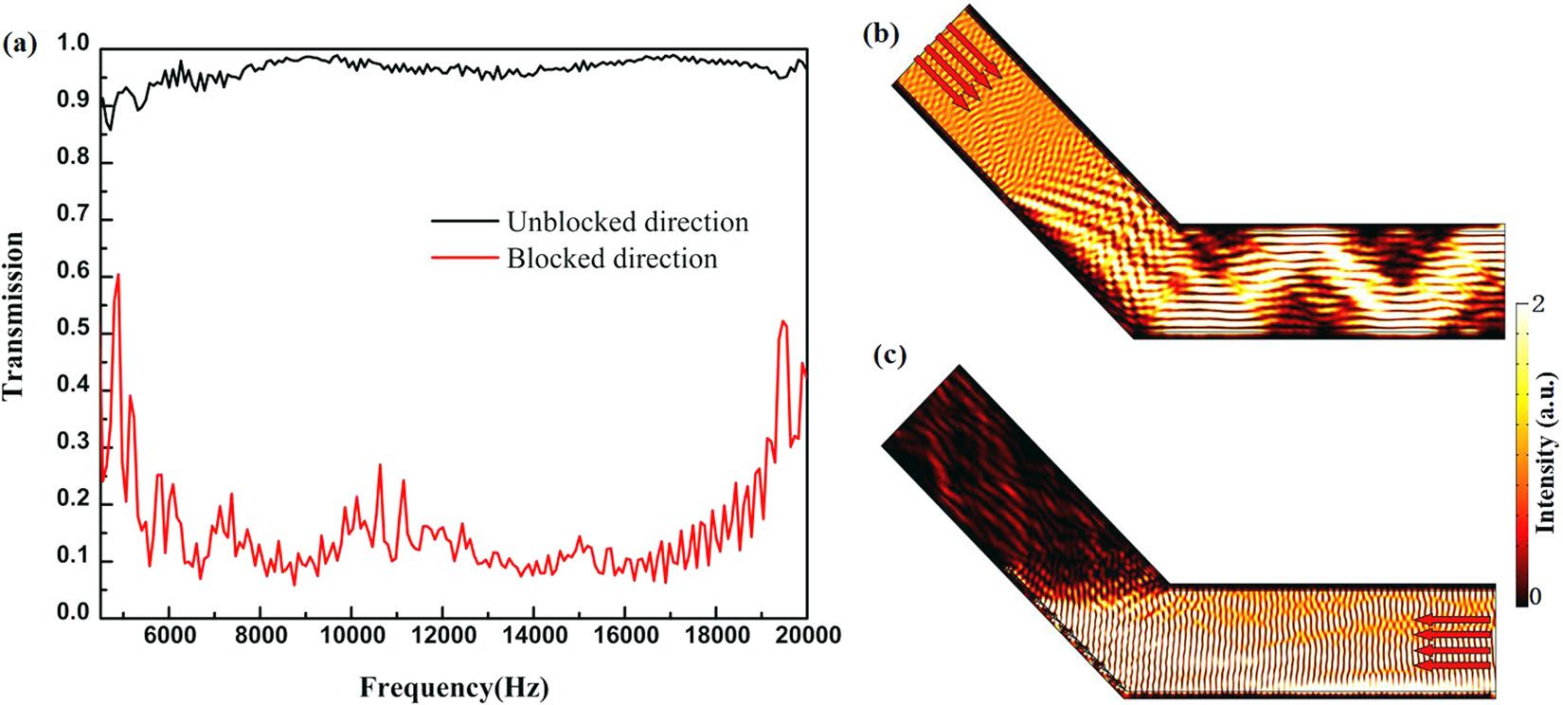
(**a**) The numerical analyses of propagation spectra for unblocked direction and blocked direction cases. The spatial acoustic intensity field distributions for (**b**) unblocked direction and (**c**) blocked direction cases at 8575 Hz, respectively. Red arrows indicate the propagation directions of the incident waves.

To verify the bandwidth performance of the designed device, the transmission for UD and BD cases is calculated from 4500 Hz to 20000 Hz and shown in Fig. 4. The transmission is simulated by calculating acoustic power along the cross-section of bend tunnel. As shown in Fig. 4(a), the remarkable AD effect is shown for comparison from 5300 Hz to19000 Hz. When the incident frequency ranges from 4500 Hz to 20000 Hz, the transmitted coefficient is more than 90% in the UD case and the transmitted coefficient is less than 30% for the BD case. Gradient acoustic metasurface can perform isolating effect of the incident acoustic wave from opposite directions. The Fig. 4(b,c) shows the AD effect of the designed model at 8575 Hz. In the UD case, the incident acoustic wave impinges on the tunnel with normal reflection, the reflected acoustic wave passes the bend tunnel, as shown in Fig. 4(b). In the case of BD, the acoustic wave impinges on the gradient acoustic metasurface, which can be held back by the negative reflection to the incident port. The gradient acoustic metasurface made of repeating supercells offers a crucial effect with the capacity of yielding broadband negative reflection.

## Discussion

In summary, this paper demonstrates an acoustic unidirectional open bend tunnel capable of realizing an acoustic analogy of electrical diode effect. The excellent characteristics results from a distinctly different mechanism of negative reflection by using acoustic gradient metasurface. We have designed an acoustic metasurface with the ability of negative reflection in an ultra-broadband frequency range and investigated the influence of incident frequency on negative reflection when the incoming angle impinging on the metasurface is over the critical incidence. Therefore, the reflective acoustic waves can be manipulated freely in a broadband frequency range by the mechanism. The proposed acoustic unidirectional bend tunnel has extraordinary properties, such as simple structure, high efficiency and ultra-broad operation bandwidth of range from 5300 Hz to 19000 Hz. This work provides a new design methodology for sound manipulation and promotes the potential applications of acoustic one-way devices such as brandband noise control, architectural acoustics and ultrasonic therapy.

## Methods

In this paper, the numerical simulation is conducted using COMSOL Multiphysics software. The steel of the model material is located in the air. The mass density and sound speed of steel are 7850 kg/m^3^ and 5100 m/s, respectively. The mass density and sound speed of air are 1.29 kg/m^3^ and 343 m/s. Perfect matched layers (PMLs) are chosen as the outer boundaries to eliminate the reflective waves.

## Electronic supplementary material

Supplementary Information

## Data Availability

All the data about this present work can reasonably be requested from Q.X.L. (liangqx728@xjtu.edu.cn).
