# Supplementary material for: Ultra-Broadband Acoustic Diode in Open Bend Tunnel by Negative Reflective Metasurface
Source: Sci Rep. 2018 Oct 31;8:16089. doi: 10.1038/s41598-018-34314-w (PMC6208409; doi:10.1038/s41598-018-34314-w)
Supplement: Supplementary file 1 — Supplementary Information [file 41598_2018_34314_MOESM1_ESM.pdf]

Supplementary Information for Ultra-Broadband Acoustic Diode in Open Bend Tunnel by Negative Reflective Metasurface

Qingxuan Liang*, Yong Cheng, Jin He, Jinke Chang, Tianning Chen, Dichen Li

School of Mechanical Engineering and State Key Laboratory for Manufacturing Systems Engineering, Xi'an Jiaotong University, Xi'an 710049, People's Republic of China

Correspondence: Qingxuan Liang (email: liangqx728@xjtu.edu.cn)

In this supplementary part, we offer the detailed information about the influence of incoming frequency on negative reflection of the gradient acoustic metasurface with different phase gradient when the incoming angle impinging on the metasurface is over the critical incidence. For the acoustic gradient metasurface with phase gradient -$\frac{\sqrt{2}}{2}k_0$, the reflection state is $n_G = -3$ with the invariant -45 ° incident angle. In the frequency range of 2000Hz to 19000Hz, there exists five possible reflection states $|n_G\rangle$: $|-1\rangle$, $|-2\rangle$, $|-3\rangle$, $|-4\rangle$ and $|-5\rangle$, corresponding the critical frequency f_{c,n_G} is 3551.9 Hz, 7103.9 Hz, 10655.6 Hz, 14207.5 Hz. When 3551.9 Hz < f < 7103.753 Hz, 7103.753 Hz < f < 10655.6 Hz, 10655.6 Hz < f < 14207.5 Hz, 14207.5 Hz < f < 19000 Hz, the corresponding reflected angle can be solved by $\theta_{re} = \arcsin[sin\theta_i + (1 + n_G)k_s \frac{f_0}{f}]$, as shown in Supplementary Fig. 1(a). The Supplementary Fig. 1(b) shows the calculated reflected acoustic felid corresponding to different incident frequencies. It can be found that when the plane acoustic wave incident at 3000 Hz, 5100 Hz, 8580 Hz, 14000 Hz,17160 Hz and 18400 Hz respectively, the beam would reflect at -45°, 29°, 45°, 36°, 45° and 38°, correspondingly. The results agree with the theoretical reflected angle of -45°, 28.8°, 44.9°, 36.3°, 44.9° and 37.7°. In the same frequency range, the possible reflection states can be deduced with the increased surface phase gradient.

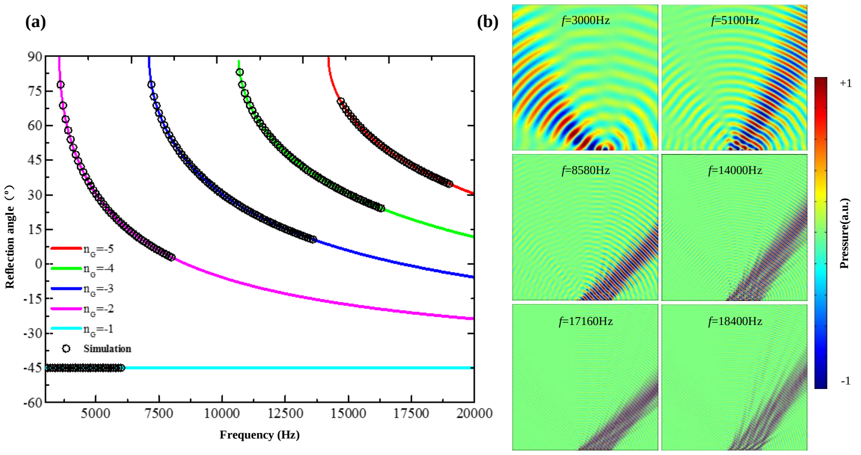

Figure S1. The reflection behavior of the gradient acoustic metasurface, when the oblique -45° plane acoustic wave incident on the phase gradient -$\sqrt{2}/2k_0$. (a) The relation between the incident frequency and the reflected angle. (b) Calculated scattered

acoustic field of different incident frequency at 3000 Hz, 5100 Hz, 8580 Hz, 14000 Hz, 17160 Hz and 18400 Hz, the beam reflect at -45°, 29°, 45°, 36°, 45° and 38°, correspondingly.

When the surface phase gradient is -1.25k_0, the reflective state is $n_G = -2$ with the invariant -45 ° incident angle. In the frequency range of 2000Hz to 19000Hz, there are three possible reflection states $|n_G\rangle$: $|-1\rangle$, $|-2\rangle$ and $|-3\rangle$. When f < 6278.9 Hz, 6278.9 Hz < f < 12557.8 Hz, 12557.8 Hz < f < 19000 Hz, the corresponding reflected angle can be solved by $\theta_{re} = \arcsin[sin\theta_i + (1 + n_G)k_s \frac{f_0}{f}]$, as shown in Supplementary Fig.2(a). Supplementary Fig.2 (b) shows the calculated reflected acoustic felid corresponding to different incident frequencies. When the oblique -45° plane acoustic wave is incoming at 4000 Hz, 8600 Hz, 10000 Hz and 17500 Hz, respectively, the beam would reflect at -45°, 32°, 21° and 31°, correspondingly. The results agree with the theoretical reflected angle of -45°, 32.6°, 21.4° and 31.1°.

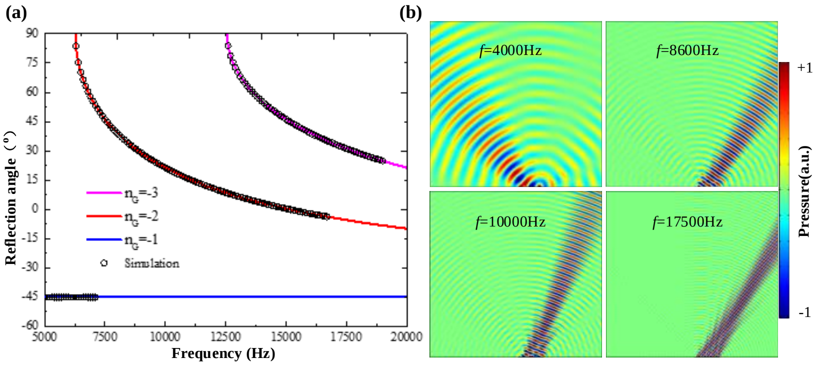

Figure S2. The reflection behavior of the gradient acoustic metasurface, when the oblique -45° plane acoustic wave incident on the phase gradient -1.25k_0. (a) The relation between the incident frequency and reflected angle. (b) Calculated scattered acoustic field of different incident frequency at 4000 Hz, 8600 Hz, 10000 Hz and 17500 Hz, the beam would reflect at -45°, 32°, 21° and 31°, correspondingly.

The results reveal the influence of incoming frequency on the possible higher order diffraction of the gradient acoustic metasurface when the incoming angle impinging on the metasurface is over the critical incidence.
